# Experience in Ceftazidime-Avibactam for treatment of MDR BGN infection in Oncologic Children

**DOI:** 10.1016/j.bjid.2025.104515

**Published:** 2025-02-21

**Authors:** Wilson Toyohiro Hoshino, Adriana Maria Paixão De Sousa da Silva, Antonio Carlos Pignatari, Ana Cristina Gales, Fabianne Carlesse

**Affiliations:** a Universidade Federal de São Paulo, Divisão de Doenças Infecciosas Pediátricas, São Paulo, SP, Brazil; b Federal Universidade de São Paulo, Instituto de Oncologia Pediátrica, Grupo de Apoio ao Adolescente e Criança com Câncer, São Paulo, SP, Brazil; c Universidade Federal de São Paulo, Divisão de Doenças Infecciosas, São Paulo, SP, Brazil

**Keywords:** Children, Pediatric, Ceftazidime-avibactam, Febrile neutropenia, Cancer, Oncology, Multiresistance, Gram negative bacilli infection

## Abstract

**Background:**

Ceftazidime-Avibactam (CAZ-AVI) plays a key role in the treatment of Multidrug Resistant Gram-Negative Bacilli (MDR-GNB) infections. In pediatrics, CAZ-AVI is clinically approved for treatment of urinary tract or intra-abdominal infection. However, there is limited data available about its use in children with cancer who have complicated infections caused by MDR-GNB.

**Objective:**

This study aims to describe our experience in using CAZ-AVI for the treatment of MDR GNB infections in children with cancer.

**Methods:**

This retrospective observational study was conducted at the Pediatric Oncology Institute (IOP/GRAACC/UNIFESP), including pediatric oncologic patients who received CAZ-AVI for the treatment of infections caused by GNB.

**Results:**

From Jan/2021 to Jun/2022, 11 patients with 13 episodes were included in the analysis. Among them, 45 % were female, with a median age of 7 years. Three patients had Acute lymphoblastic Leukemia (ALL), three had Acute Myeloid Leukemia (AML), two had Non-Hodgkin Lymphoma (NHL). Additionally, there was one case each of medulloblastoma, fibrosarcoma, and craniopharyngioma. All patients presented significant risk factors for MDR-GNB, such as neutropenia and two were submitted to Hematopoietic Stem Cell Transplantation (HSCT). The infection episodes included six Bloodstream Infections (BSI), two Urinary Tract Infections (UTI), two tracheobronchitis cases, along with one case each of necrotizing pneumonia, ventriculitis, and endocarditis. The identified pathogens included *Klebsiella pneumoniae, Pseudomonas spp*., *Enterobacter cloacae*, and *Stenotrophomonas maltophilia*. The primary reason for prescribing CAZ-AVI was either Multidrug-Resistant Gram-Negative Bacteria (MDR-GNB) infection or clinical worsening after initial therapy. Combination therapy was prescribed in eight episodes with a median prescription length of nine days. Microbiological sterilization was achieved in 92 % of episodes, and the 30-day survival rate was 84 %. Notably, no deaths were associated with treatment failure, and no adverse events associated with CAZ-AVI use were observed.

**Conclusion:**

CAZ-AVI could be used for treating GNB infections in oncologic pediatric patients.

## Introduction

Cancer patients undergo some of the most intense treatments in medicine, including myelosuppressive chemotherapy drugs, radiotherapy and Hematopoietic Stem Cell Transplantation (HSCT). Consequently, these patients, particularly in the pediatric population, become highly vulnerable to the most significant and life-threatening complication: the invasive infections.^1^

Neutropenia coupled with fever may be the first signs of a life-threatening infection. Once the diagnosis of Febrile Neutropenia (FN) has been made, broad-spectrum intravenous antibiotic therapy is empirically prescribed for these patients^2^, ^3^, ^4^ decreasing mortality from invasive infections from 90 % in the 1970s^5^ to below 1 % in the years 2000.^6^ With increased survival, these patients now experience two or more episodes of FN during cancer treatment. Over the last decade, there has been an estimated increase of more than 50 % in the number of hospitalizations for febrile neutropenia.^6^ Consequently, patients with this condition often undergo prolonged and repeated courses of antibiotic therapy. When coupled with factors such as the use of prophylactic antibiotics, multiple and extended hospital stays, care in the Intensive Care Unit (ICU) and the use of invasive devices, these circumstances pose significant risks for the emergence of Multidrug-Resistant (MDR) bacterial infections.^7^^,^^8^

Recently, an increase in the BSI caused by Gram-Negative Bacilli (GNB) has been observed, specifically those caused by Multidrug-Resistant (MDR) which are associated with an increased demand for intensive care, a higher likelihood of progressing to severe conditions and greater morbimortality^6^^,^^9^ making the better understanding of its management and development of new therapies urgent.^10^

In pediatric onco-hematological patients, the treatment of MDR infections poses greater challenges, including a limited array of therapeutic options, insufficient data on pharmacokinetics/pharmacodynamics, and concerns about drug safety, particularly in the context of chemotherapy regimens and in critical clinical conditions.

To date, in low- and middle-income countries such as Brazil, polymyxins in combination are still the most prescribed antibiotic for treatment of carbapenemase-producing Enterobacterales infections due to its lower cost and greater availability.^11^ However, the use of polymyxins has been associated with severe adverse events such as nephrotoxicity up to 70 % and neurotoxicity in 3 %.^12^ In addition, drug interactions and allergic reactions leading to interruption and therapeutic failure are commonly observed especially in oncologic patients, who receive many other therapeutic agents.^13^^,^^14^ For these reasons, safer and more effective antimicrobials as ceftazidime-avibactam, meropenem-vaborbactam and imipenem-relebactam have been recommended by European and American guidelines as the first choice for treatment of carbapenem-resistant GNB.^15^^,^^16^

In Brazil, among the new therapeutic options, ceftazidime-avibactam is the only approved option for pediatric patients. The Brazilian National Health Surveillance Agency (Anvisa) granted approval for Ceftazidime-Avibactam (CAZ-AVI) for children over 3 months to 18 years of age in 2020 for urinary tract and intra-abdominal infection. While studies in the pediatric population have demonstrated the safety and efficacy of the drug, there is a notable scarcity of research focused on pediatric oncologic patients.

To date, there are only two case series in children with cancer.^17^^,^^18^ This case series aimed to describe the use of ceftazidime-avibactam to treat other complicated infections caused by GNB children undergoing antineoplastic treatment and Hematopoietic Stem Cell Transplantation (HSCT) in Brazil. This population diverges from phase two studies^19^^,^^20^ because they are critically ill, due to the infection severity and present comorbidities related to cancer.

## Methods

This is a retrospective observational study that includes all pediatric cancer patients hospitalized at the Pediatric Oncology Institute (IOP/GRAACC/UNIFESP), who received CAZ-AVI for the treatment of GNB infections between 01 January 2021 and 30 June 2022.

IOP is a pediatric oncology hospital recognized as a leading institution in Latin America. It has 56 beds with 10 intensive care unit beds, treating approximately 430 new cancer cases annually and conducting around 80 bone marrow transplants each year. The institute supports a technical and scientific partnership with the Universidade Federal de São Paulo. The IOP Infection Prevention and Control team (IPC) diligently checks all GNB infections within the facility, documenting all cases of infections data. The cases described in this study were identified by the IPC database. The data collection was obtained through review of clinical charts.

The study included children and adolescents patients aged between 3-months and 18-years with cancer who underwent or not to HSCT receiving CAZ-AVI for more than 72-hours to treat documented GNB infection. The 3-day period is commonly used in intention to treat analysis and is important to assess the choice and review the severity of the illness if the patient's condition worsens or there's no improvement in a antimicrobial stewardship program.^21^

Infection was defined by the presence of BGN associated with one or more clinical symptoms. Patients whose clinical data could not be obtained or had been in use of CAZ-AVI for less than three days of treatment or empirical use were excluded.

Treatment success was defined as resolution of signs and symptoms related to the infection, microbiologic negative control and improvement of laboratory test results at the end of therapy course. Clinical failure was defined as laboratory and clinical worsening of the symptoms that required additional intervention as therapy change due to infection.

Patients were followed for 30-days, and a clinical form was completed, capturing demographic and clinical information, underlying disease, history of HSCT, use of antibiotic prophylaxis, prior antimicrobial treatments, history of previous surgery, presence of indwelling devices, earlier MDR GNB colonization, time to cultures become negative, details of empirical and definitive therapy, mortality at 14- and 30-days. Additionally, concurrent infections with potential impact on clinical outcomes were assessed. Laboratory data, including neutrophil count, C-Reactive Protein (CRP), urea, creatinine, Alanine Aminotransferase (ALT) and Aspartate Aminotransferase (AST) were also evaluated.

### *Microbiological assessment*

All collected cultures underwent processing at the routine microbiology laboratory of the Central Laboratory of Hospital São Paulo (EPM/UNIFESP). For the isolates recovered from the positive blood cultures, Identification of bacterial isolates at the species level and antimicrobial susceptibility testing was initially performed by the automated BD Phoenix™ method (Becton Dickinson, Microbiology Systems, MD). For specimens other than blood, species identification was performed by MALDI-TOF MS using the Microflex LT mass spectrometer and Biotyper 3.3 software (Bruker Daltonics), according to the manufacturer's recommendations. Susceptibility profiles were determined using disk diffusion. Polymyxin B susceptibility testing, irrespective of the clinical specimen, was determined by broth microdilution following ISO 20,776–1:2019 standards and interpreted per BrCAST/EUCAST guidelines.^22^

MDR BGN isolate was defined as resistant to at least three classes of antimicrobials following the 2012 International consensus.^23^

The phenotypic detection of carbapenemase by Blue-Carba test as well as confirmation of carbapenemase-encoding genes was achieved through PCR at the research facility, Laboratório Alerta, Division of Infectious Diseases, UNIFESP. The primers targeted *bla*_KPC,_ *bla*_NDM_, *bla*_OXA 48_, *bla*_SPM_, *bla*_IMP_, *bla*_SIM_, *bla*_VIM_, and *bla*_GIM_, as previously described.^24^^,^^25^

### Treatment

Patients received CAZ-AVI for a minimum of 72 hours, either as monotherapy or in combination. The standardized dose used was 50mg/kg/dose of ceftazidime 8/8h (62,5 mg/kg/dose of CAZ-AVI), maximum dose of 2g (2.5g of CAZ-AVI), administered over a two-hour infusion. CAZ-AVI doses were adjusted according to estimated glomerular filtration rate, considering the use of renal replacement therapy.

We evaluated resolution of clinical signals and symptoms, time to fever defervescence, presence of allergic reactions (rash, angioedema, anaphylaxis), time to cultures become negative, laboratory records including blood counting and neutrophil recovery time, C-reactive protein, kidney and liver function. Mortality at 14- and 30-days was also recorded. Therapeutic failure was defined as clinical and laboratory deterioration after at least 72h of empirical therapy.

The study was approved by the IOP/GRAACC and scientific committee. Registration was carried out with the UNIFESP Research Ethics Committee and Plataforma Brasil (CAAE n°67,434,523.1.0000.5505). The analysis was carried out and the findings were displayed in absolute frequency and percentage in a descriptive way.

## Results

Our analysis included thirteen episodes of GNB infections in eleven patients treated with CAZ-AVI. Notably, two patients experienced two episodes of infections more than thirty days apart. Among the patients, five patients were female (45 %), with an average age of 7 years (ranging from 4 to 16-years of age, mode of 4-years). The oncological diagnoses encompassed three cases of Acute Lymphoid Leukemia (ALL), three cases of Acute Myeloid Leukemia (AML), two cases of Non-Hodgkin's Lymphomas (NHL), and 3 solid tumors (23.0 %). Allogeneic HSCT was performed for two AML patients. In 10/13 (76 %) of the episodes, the patient was neutropenic when the infectious diagnosis was made and treatment with ceftazidime-avibactam was started. Relevant comorbidities were detailed in Table 1.

**Table 1:** Demographic data and risk factors of the patients with GNB infection included in the study.

Pct.	Ep.	Age	Gender	Oncological diagnosis	HSCT	Neutropenia	Devices / Support	Comorbidity	Previous colonization / MDR GNB infection	Prior antibiotic
1	1	4	M	ALL	No	Yes	PAC	Parainfluenza Infection	Rectal Swab CR KP	Meropenem, imipenem polymyxin B
2	2a	4	M	ALL	No	Yes	PAC / PICC	No	CR-KP UTI	Polymyxin B, meropenem, amikacin
	2b	4	M	ALL	No	Yes	PAC / PICC	No	CR-Pseudomonas spp. BSI	Polymyxin B, CAZ-AVI, meropenem, amikacin
3	3	9	M	ALL	No	No	CVC, PAC	*Acremonium persicinum* infection in treatment	Feces: CR-PA	Meropenem, amikacin, levofloxacin, polymyxin B
									Rectal Swab: CR-KP	
4	4	6	F	AML	No	Yes	PAC	Reaction to vancomycin, meropenem and polymyxin B infusion	No	Amikacin, PTZ, meropenem, polymyxin B
5	5	12	F	AML	Allogeneic HSCT	Yes	CVC, MV, Hemodialysis	Noonan syndrome, 22 triploid	No	Cefepime, meropenem
6	6	16	F	AML	Allogeneic HSCT	Yes	PAC, CVC, MV	Previous SARS-CoV-2 + MISC with coronary dilation	No	Meropenem, amikacin
7	7	4	F	NHL	No	Yes	PAC +VPS	CNS invasion + VPS	Rectal swab: Klebsiella spp. ESBL	Cefepime, amikacin and vancomycin
8	8	8	M	NHL	No	Yes	CVC, PAC	Amoxicillin allergy	No	Cefepime, amikacin, metronidazole
9	9	4	F	Medulloblastoma	No	Yes	EVPS	Hydrocephalus with PVD	Ventriculitis / UTI by CR KP	Meropenem, amikacin
10	10a	7	M	Craniopharyngioma	No	No	CVC, PAC, EVD	Panhypopituitarism + VPS	No	Meropenem, vancomycin
	10b	7	M	Craniopharyngioma	No	No	PAC, VPS	Panhypopituitarism + VPS	CR-KP Ventriculitis	CAZ-AVI, meropenem
11	11	8	F	Infantile-type fibrosarcoma	No	Yes	CVC, PAC	Pulmonary and CNS metastasis	No	Polymyxin B

M, Male; F, Female; ALL, Acute Lymphocytic Leukemia; AML, Acute Myeloid Leukemia; NHL, Non-Hodgkin's Lymphoma, HSCT, Hematopoietic Stem Cell Transplant; CVC, Central Venous Catheter; PAC, Port A Cath; PICC, Central Catheter Peripheral Insertion; MV, Mechanical Ventilation; EVD, External Ventricular Drain; EVPS, External Ventriculoperitoneal Shunt; VPS, Ventriculoperitoneal Shunt; MISC, Multi Inflammatory Syndrome in Children; Kp, *Klebsiella pneumoniae*; PA, *Pseudomonas Aeruginosa*; CR, Carbapenem Resistant; CNS, Central Nervous System; IT, Intrathecal; ESBL, Extended Spectrum Beta Lactamase; PTZ, Piperacillin-Tazobactam; UTI, Urinary Tract Infection; GNB, Gram Negative Bacteria; CAZ-AVI, Ceftazidime-Avibactam.

### *Colonization and previous infection*

Among the 11 patients, 7 (63 %) had rectal colonization by MDR-GNB. *Klebsiella* spp. was the most frequent agent found colonizing three patients (two carbapenemase-producing isolates and one ESBL producer). A single patient showed rectal colonization by both carbapenemase-producing *K. pneumoniae* and carbapenem-resistant *P. aeruginosa*. Four patients had a significant history of earlier MDR GNB infections: two patients had carbapenem-resistant *Klebsiella Pneumoniae* (CR-KP) ventriculitis, one Urinary Tract Infection (UTI) caused by CR-KP, and one BSI caused by *Pseudomonas* spp.

### *Characteristics of infections*

BSI (46.0 %; 6 cases) was the most frequent infection site followed by UTI, tracheitis (2 cases each), one case of tracheitis was associated with secondary BSI. Pneumonia, ventriculitis and endocarditis (one case each).

All episodes were associated with clinical signs and/ or laboratory worsening at the moment of microbiologic BGN identification. It's this study all patients were immunosuppressed either neutropenic or critically ill.

Among the thirteen episodes of infection, seven were caused by *Klebsiella pneumoniae,* three by *Pseudomonas spp*. (2 *Pseudomonas aeruginosa* and 1 *Pseudomonas* spp.), two by *Enterobacter cloacae* and one by *Stenotrophomonas maltophilia*. Clinical, laboratory and therapeutic characteristics are depicted on Table 2.

**Table 2:** Clinical, laboratory and therapeutic characteristics of the infection episodes included in the study.

Pct.	Ep.	Oncological diagnosis	Infection	Agent	Definitive therapy	Duration of treatment (days)	Negativation	ICU	Device / Support	Outcome 30-days
1	1	ALL	BSI	*Pseudomonas aeruginosa*	CAZ-AVI	8	D5	Yes	MV, VAD	Death 10 days
2	2a	ALL	BSI	*Pseudomonas* spp.	CAZ-AVI + meropenem + amikacin	14	D7	Yes	MV/ CVC	Survival
	2b	ALL	BSI	*Klebsiella pneumoniae*	CAZ-AVI	7	D10	Yes	No	Survival
3	3	ALL	Tracheitis	*Klebsiella pneumoniae*	CAZ-AVI + gentamicin	14	D10	Yes	MV, VAD	Survival
4	4	AML	BSI	*Klebsiella pneumoniae*	CAZ-AVI + amikacin	7	D4	No	No	Survival
5	5	AML	Pneumonia	*Pseudomonas aeruginosa*	CAZ-AVI + aztreonam + meropenem	14	NO^b^	Yes	MV, VAD Hemodialysis	Survival
6	6	AML	Tracheitis + 2° BSI	*Stenotrophomonas maltophilia*	CAZ-AVI + aztreonam	22	D2	Yes	MV, TQT, VAD	Death 22 days
7	7	NHL	BSI	*Klebsiella pneumoniae*	CAZ-AVI + meropenem + amikacin	14	D7	Yes	MV	Survival
8	8	NHL	Endocarditis	*Enterobacter cloacae*	CAZ-AVI + aztreonam + meropenem	14	D3	Yes	No	Survival
9	9	Medulloblastoma	BSI	*Enterobacter cloacae*	CAZ-AVI	14 days	D3	Yes	EVPS	Survival
10	10a	Craniopharyngioma	Ventriculitis	*Klebsiella pneumoniae*	CAZ-AVI + meropenem + levofloxacin	21	Maintained^a^	Yes	TWO	Survival
	10b	Craniopharyngioma	UTI	*Klebsiella pneumoniae*	CAZ-AVI	15	D5	Yes	No	Survival
11	11	Infantile-type fibrosarcoma	UTI	*Klebsiella pneumoniae*	CAZ-AVI	7	D5	No	No	Survival

ICU, Intensive Care Unit; MV, Mechanical Ventilation; VAD, Vasoactive Drug; EVD, External Ventricular Derivation; TQT, Tracheostomy; EVPS, External Ventriculoperitoneal Shunt; UTI, Urinary Tract Infection; BSI, Bloodstream Infection; GIT, Gastrointestinal Tract; CAZ-AVI, Ceftazidime-Avibactam; S, Survival; D, Death.

a Culture turned negative after initial therapy and maintained during CAZ-AVI use; ^b^ Positive bronchoalveolar lavage culture persisted, considered colonization.

The antimicrobial susceptibility profile of the bacterial isolates responsible for causing infections was displayed in Table 3. All seven *K. pneumoniae* isolates were resistant to meropenem, ciprofloxacin and cefepime. In contrast, four and five isolates were susceptible to polymyxin B and aminoglycosides, respectively. Among the seven *K. pneumoniae*, only three were available for further microbiological characterization. *Klebsiella pneumoniae* carbapenemase encoding gene was found in three isolates. One *E. cloacae* (episode #9) isolate was susceptible to meropenem, amikacin, ciprofloxacin and cefepime, while the other *E. cloacae* isolate was susceptible only to polymyxin B. No carbapanemase encoding gene was detected in both *E. cloacae* isolates. The two *P. aeruginosa* isolates showed resistance to all antimicrobials tested, except polymyxin B and amikacin (one isolate only). Both *P. aeruginosa* were identified as producers of SPM-1, a class B carbapenemase. The *Pseudomonas* spp. isolate was susceptible only to amikacin, while *Stenotrophomonas maltophilia* isolate showed susceptibility in vitro to levofloxacin: MIC (0.25 µg/mL) and tigecycline: MIC (0.016 µg/mL) (Table 3).

**Table 3:** Antimicrobial susceptibility profile of bacterial isolate according to episode of infection.

Pct.	Ep.	Site	Agent	Susceptible	Susceptible, increased exposure	Resistant
1	1	BSI	*Pseudomonas aeruginosa*	Amikacin, polymyxin B	‒	Cefepime, ceftazidime, meropenem, ciprofloxacin, PTZ, imipenem
2	2a	BSI	*Pseudomonas* spp*.*	Amikacin	Cefepime, ceftazidime, ciprofloxacin, PTZ, imipenem	Meropenem, polymyxin B
	2b	BSI	*Klebsiella pneumoniae*	Amikacin, gentamicin, polymyxin B	‒	Cefepime, ceftriaxone, ceftazidime, meropenem, ertapenem, ciprofloxacin, TMP/SMX
3	3	Tracheitis	*Klebsiella pneumoniae*	Gentamicin	Amikacin	Cefepime, ceftazidime, ceftriaxone, ciprofloxacin ertapenem, meropenem, PTZ, polymyxin B (mic>8)
4	4	BSI	*Klebsiella pneumoniae*	Amikacin, polymyxin B	‒	Cefepime, ceftazidime, gentamicin, ciprofloxacin, PTZ, imipenem
5	5	Pneumonia	*Pseudomonas aeruginosa*	Polymyxin B	‒	Amikacin, cefepime, ceftazidime, ciprofloxacin, imipenem, PTZ, meropenem
6	6	BSI + tracheitis	*Stenotrophomonas maltophilia*	Levofloxacin, tigecycline	‒	ND
7	7	BSI	*Klebsiella pneumoniae*	Amikacin	‒	Cefepime, ceftazidime, ceftriaxone, ciprofloxacin ertapenem, meropenem, PTZ, polymyxin B
8	8	Endocarditis	*Enterobacter cloacae*	Polymyxin B	‒	Amikacin, cefepime, ceftazidime, gentamicin, meropenem, ceftriaxone, ciprofloxacin, PTZ, ertapenem
9	9	BSI	*Enterobacter cloacae*	Meropenem, amikacin, ciprofloxacin	Cefepime	Ceftazidime, amikacin, ceftriaxone, PTZ, ertapenem, Polymyxin B
10	10a	Ventriculitis	*Klebsiella pneumoniae*	Amikacin, gentamicin, polymyxin b	‒	Cefepime, ceftazidime, ceftriaxone, ciprofloxacin, ertapenem, meropenem, PTZ
	10b	UTI	*Klebsiella pneumoniae*	Polymyxin B	‒	Amox-Clav, ceftriaxone, cefepime, ertapenem, meropenem, ciprofloxacin, amikacin
11	11	UTI	*Klebsiella pneumoniae*	‒	‒	Amox-Clav, cefepime, ceftriaxone, cefuroxime, ciprofloxacin, TMP/SMX, ertapenem, meropenem, amikacin, polymyxin B

BSI, Bloodstream Infection; UTI, Urinary Tract Infection; TMP-SMX, Trimethoprim-Sulfamethoxazole; PTZ, Piperacillin-Tazobactam, Amox-Clav, Amoxicillin-Clavulanate; NA, Not Available; KPC, KPC gene; AmpC, AmpC phenotypic; SPM, SPM gene; Ind., Indeterminate.

^a^Susceptibility profile was determined using BrCAST/EUCAST breakpoints; ^b^ SPM-1 encoding gene was detected in both isolates.

### Treatment

The reasons for using CAZ-AVI included contraindications to polymyxin B (46 %), polymyxin B resistance (30 %), and clinical worsening with the first regimen (23 %). In episode #9, the *E. cloacae* isolate was sensitive to meropenem, but presented fever and CPR elevation in use of meropenem and amikacin and was decided to escalate to CAZ-AVI with good response.

CAZ-AVI was used in 5 episodes (38 %) as monotherapy and in 8 episodes (61 %) as part of combination therapies with gram-negative coverage. Among these, it was paired with an aminoglycoside in two instances and with both an aminoglycoside and meropenem in two others. Additionally, a combination with aztreonam was employed in three episodes to cover metallo-beta-lactamase-producing Gram-Negative Bacilli (GNB), including *S. maltophilia* infections. Meropenem was also associated in two of those cases. In one episode, a combination of meropenem and levofloxacin was used. The most common combination involved meropenem in five episodes. These antimicrobial associations were kept as salvage therapy. The average time of use of CAZ-AVI was 9 days, ranging from 7 to 22 days.

Among the 13 episodes, 11 (84 %) needed Intensive Care (ICU) during treatment. Eight (61 %) were considered critical patients, requiring mechanical ventilation and vasoactive drugs. In 10 of the episodes, patients had an increase in the estimated glomerular filtration rate above 130 mL/min/m^2^ (Schwartz Method up to 16-years-old/CKD-EPI for over 16-years-old) before the initiation of treatment. Additionally, one patient needed renal replacement therapy due to clinical deterioration, and dose adjustment was implemented.

All patients had received a broad-spectrum antibiotic therapy regimen for GNB in the 30 days preceding CAZ-AVI use. Ten patients had previously received meropenem, and four had received polymyxin B. Other antibiotics used included cefepime and piperacillin with tazobactam (Table 1).

In seven of 13 (53 %) of the episodes, the patient was neutropenic when the infectious diagnosis was made and treatment with CAZ-AVI was started.

In the episode of ventriculitis, despite negative cultures, the patient continued to have neurological symptoms and cytological and biochemical parameters compatible with infection, a significant improvement was observed on the third day of using CAZ-AVI.

In episode 6, it was not possible to perform an antibiogram of the *Stenotrophomonas maltophilia* isolate. Despite initial treatment with polymyxin B the treatment failed, however, there was improvement after starting the CAZ-AVI and aztreonam in synergy.

In episode 9, despite the identification of *E. cloacae* susceptible to meropenem and cefepime, the patient clinically deteriorated after treatment with meropenem, and CAZ-AVI was indicated with a good clinical response.

### *Clinical and laboratory evolution*

**Fig. 1:**
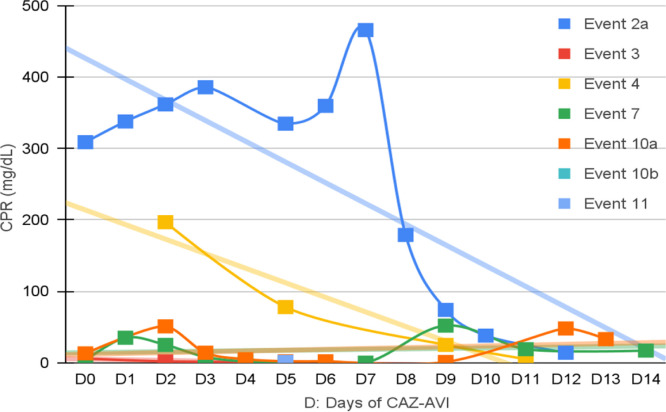
CPR level in *K. pneumoniae* cases.

**Fig. 2:**
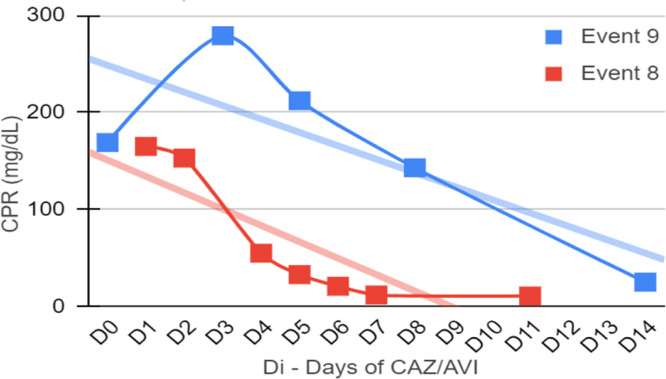
CPR level in *E. cloacae* cases.

**Fig. 3:**
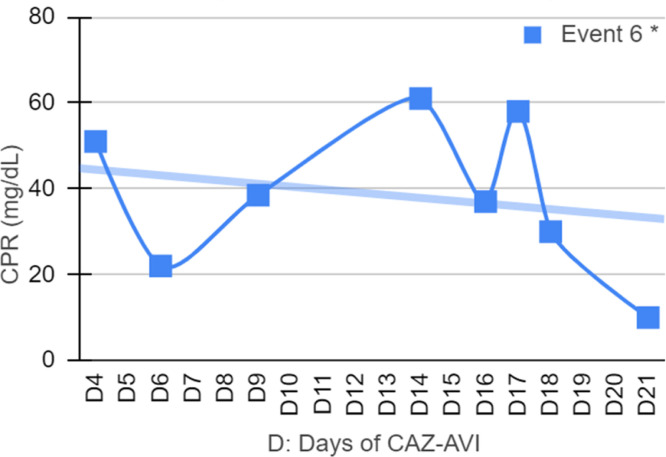
CPR level in *S. maltophilia.*

**Fig. 4:**
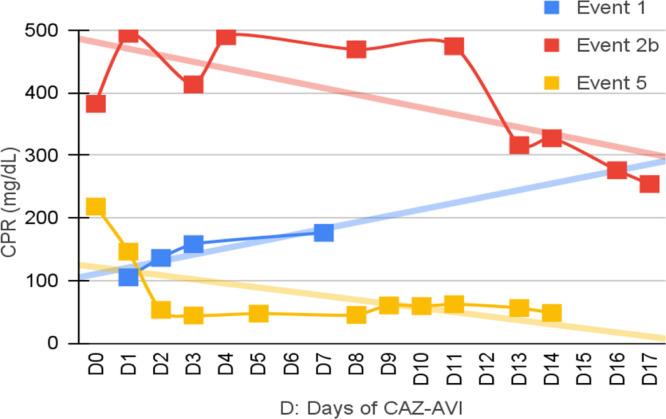
CPR level in *Pseudomonas spp*. cases.

Seven patients were neutropenic when CAZ-AVI was prescribed. Four of them remained neutropenic during GNB infection treatment. Only in episode #1, the patient persisted neutropenic and perished. In three episodes patients resolved the neutropenia by day 12th, 5th and 5th of treatment with clinical improvement and CPR demotion (Fig. 1, Fig. 2, Fig. 3, Fig. 4).

No fever was observed in six episodes. Among the seven episodes associated with fever, the time to become afebrile varied from one to four days, with the most common being within 72 hours (80 %). Four of these episodes occurred in neutropenic patients. In two episodes the fever persisted until Day 10 and Day 16 despite the antibiotic treatment leading to patient death.

Fig. 1, Fig. 2, Fig. 3, Fig. 4 illustrates the CRP evolution during the infection episode and CAZ-AVI treatment. Notably, only one episode exhibited a tendency for increased CRP during treatment, and unfortunately, this patient succumbed due to the progression of the oncological disease. In six episodes, a continuous drop in CRP was seen shortly after the introduction of CAZ-AVI. In three episodes, there was an increase up to 72 hours after the start of the antimicrobial with a progressive decrease afterwards and in another three there was an increase in CRP during treatment between D7 and D14 with a decrease afterwards. Patient number 7 presented a progressive drop in CRP to 10 mg/dL but died due to massive gastrointestinal bleeding. Growth of *Trichosporon asahii* in blood cultures was identified after his death.

In five episodes, hypoalbuminemia (<3g/dL) was observed before CAZ-AVI prescription, which was resolved during treatment of three cases. Albumin measurement was not performed in three episodes.

### *Clinical and microbiological outcomes*

Negative infection site cultures were achieved in 12 of 13 episodes (92 %), all were reached at the first control culture. For BSI cases, negativity was documented between 3 and 10 days, with a median of 6 days. In the endocarditis case, blood culture became negative 48 hours after treatment initiation. In urinary tract infections, microbiological negativity was achieved after seven and ten days, while in tracheitis, cultures turned negative after 30 days.

In ventriculitis, cultures remained negative, accompanied by a significant improvement in the clinical presentation and notable improvements in biochemical and cytological CSF parameter's post- introduction of CAZ-AVI. In a necrotizing pneumonia episode caused by *Pseudomonas aeruginosa*, positive bronchoalveolar lavage cultures persisted after four months of clinical improvement and discontinuation of therapy, considered as colonization. In episode number 9, despite the isolation of *E. cloacae* being susceptible to meropenem and cefepime, the patient experienced clinical worsening without other microbiological evidence. Improvement was observed after escalating treatment to CAZ-AVI.

The study cohort experienced two deaths, resulting in a survival rate of 93 % at 14 days and 84 % at 14 days 30 days One patient expired due to progression of hematological disease with negative cultures, and another patient died due to massive gastrointestinal bleeding, with cultures negative for GNB under treatment, but positive for *Trichosporon asahii* in peripheral blood culture.

### *Adverse events*

No serious adverse events related to the use of CAZ-AVI were seen in any of the episodes. Two patients (episodes 2a and 5) had an increase in liver transaminases (twice the normal value) at the beginning of CAZ-AVI treatment, which improved during treatment. In three episodes we observed an increase in transaminases during the use of the medication, with subsequent normalization as clinically improvement occurred. No dermatological reactions were identified.

## Discussion

The World Health Organization (WHO) estimates that multidrug resistant microorganisms will be responsible for top 3 leading causes of death by 2050 killing up to 10 million people annually.^26^ This raises even greater concern in high-risk populations such as pediatric oncology patients.

Agud et al. evaluated the risk factors for colonization by MDR in children with complex conditions in Spain and found that the main risk factors were use of prophylactic antibiotics, use of immunosuppressive medication, skin lesions, greater number of surgeries and prolonged stay in hospital in the last 12-months.^27^ Patients included in this study had all risk factors for MDR described in the literature such ICU stay, previous use of broad-spectrum antibiotics, skin wound, invasive devices and colonization with MDR GNB.

In this study, the average age was 7 years with a prevalence of hematological diseases such as ALL, AML and NHL (72 %), compatible with literature data on MDR GNB infections in pediatric oncology patients.^28^ In a Brazilian study, Costa et al. found that MDR GNB infections in oncology children had a mean age of 7 years and hematological disease and healthcare-associated infection were also risk factors.^29^ Casseli et al. observed in a multicenter study with oncology children a higher average age of 7 years at infection when associated with *Pseudomonas aeruginosa* MDR compared to non-MDR *Pseudomonas* spp. of 4 years of age. They also found a higher prevalence of hematological diseases up to 70 % in infections caused by *Pseudomonas spp*.^28^

In this study, the prescription of CAZ-AVI was primarily due to MDR GNB infection, especially to those resistant to carbapenemases, secondly to therapeutic failure of the first regimen with good clinical response.

The main site of infection was the bloodstream, with blood cultures showing negative results in all BSI cases at the first control test. A systematic review and meta-analysis support the security and efficacy of CAZ-AVI in BSI due to carbapenem-resistant Enterobacteriaceae finding lower 30-day mortality and lower nephrotoxicity when compared to other antibiotic regimens such as colistin.^30^

This study highlights that pediatric oncology patients present peculiarities such as an important history of colonization and previous MDR infection, use of broad-spectrum antibiotics during prolonged hospitalizations, progression to severe conditions requiring extended intensive care unit stays.

This population is deeply immunocompromised by baseline disease, chemotherapy, neutropenia or in critical ill status that may lead to no classical symptoms of infection. The presence of fever, clinical alteration and laboratory changes may be the only signs of infection.^31^

In view of this, the support of microbiology to prompt identification of the infectious agent as well as its resistance mechanisms and phenotypes becomes of foremost importance, enabling the adequate targeted therapy.

Among the isolated agents, the most prevalent were Enterobacteriaceae, *Klebsiella pneumoniae* and *Enterobacter cloacae* carbapenem resistant. The resistance to carbapenems in Enterobacteriaceae is generally due to the production of enzymes Class A Serine Carbapanemase type, with KPC-2 being the most prevalent in Brazil. According to a national report, since 2020, an increase in the frequency of NDM producer isolates and NDM/KPC co-producers has been documented, accelerated by the COVID-19 pandemic.^32^

CAZ-AVI was also prescribed for infections caused by *Pseudomonas* spp. and *Stenotrophomonas maltophilia*. In addition to the enzymatic resistance mechanisms, antimicrobial resistance in non-fermenting gram-negative bacilli has specific characteristics that make their treatment particularly challenging.

*Pseudomonas aeruginosa* stands out for its ability to acquire multiple resistance mechanisms and is classified as Difficult to Treat (DTR) by the CLSI criteria when they present a phenotype of resistance to first-line antimicrobials: ceftazidime, aztreonam, cefepime, piperacillin-tazobactam, imipenem, meropenem and quinolones. Its resistance can be explained by mutation of its ampC, PDC (Pseudomonas-Derived Cephalosporinases), hyperproduction of PDC, increase in the efflux system due to increase in MexAB-OprM and entry deficiency due to porin reduction OprD, in addition to the acquisition of enzymes such as metallocarbapenemases.^32^^,^^33^

In the study, *Pseudomonas spp*. BSI showed resistance solely to meropenem and polymyxin B. This finding suggests a non-aeruginosa *Pseudomonas* infection, since the colistin resistance may be intrinsic in *Pseudomonas fluorescens* complex (e.g., *P. chlororaphis* or *P. koreensis)* or adaptive by genes emrA, lpxA, lpxD, pgsA, phoP and phoQ leading to the addition of cationic compounds such as L-ara-4n to the lipopolysaccharide of the outer bacterial membrane.^34^ It was not possible to identify the species of *Pseudomonas* found and the possibility of *Pseudomonas aeruginosa* infection is unlikely since the prevalence of resistance to colistins by *P. aeruginosa* is low, less than 2 %.^35^ The *P. aeruginosa* strains presented an indeterminate Blue-Carba test and a positive blaSPM-1 gene test. The Blue Carba test demonstrated a sensitivity of 93.6 % and a specificity of 100 % for detecting carbapanemase producing *Pseudomonas* spp*.* However, this text has shown important limitations in detecting class B carbapanemase. Although the frequency of SPM-1 producing *P. aeruginosa* has declined over the years in Brazilian centers, *P. aeruginosa* carrying *bla*SPM gene are still recovered in our hospital, where it was firstly reported.^36^

Case number 6, the patient presented an invasive infection by *Stenotrophomonas maltophilia*, a non-fermenting GNB in the bloodstream and tracheal aspirate culture. Risk factors for this infection included invasive devices, urinary tract infection, and extensive prior antimicrobial use, as meropenem and aminoglycosides. Notably, the patient had a history of severe SARS-CoV-2 infection that progressed to MIS-C (Multi-Inflammatory Syndrome in Children)^37^ with coronary dilation. Secondary infection by *S.* maltophilia was recurrent complication associated to SARS-CoV-2 infection during the pandemic.^38^

For severe *S. maltophilia* infections, the Infectious Diseases Society of America (IDSA) recommends combination therapy with first-line antibiotics such as levofloxacin, minocycline, sulfamethoxazole-trimethoprim, and ceftazidime. *Stenotrophomonas maltophilia* exhibits resistance to carbapenems and other beta-lactams due to the presence of Serine beta-lactamases L2, conferring an ESBL-like resistance profile. Additionally, resistance to aztreonam can occur through the production of metallo-beta-lactamase L1. Furthermore, *S. maltophilia* possesses RND, MFS, and ABC efflux systems, enabling resistance to aminoglycosides, quinolones, tetracyclines, macrolides, and chloramphenicol.^39^

Ceftazidime and avibactam, while active against serine carbapenemases, are inactivated by metallo-beta-lactamase. On the other hand, aztreonam is a monobactam that is not hydrolyzed by metallo beta lactamases, but is inactivated by extended spectrum betalactamases and serinecarbapenemases. This complementary activity enables effective combination therapy against the increasingly prevalent bacteria that co-produce metallo-beta-lactamases and extended-spectrum serine beta-lactamases. Consequently, the combination of ceftazidime-avibactam with aztreonam emerged as a therapeutic possibility. Furthermore, the aztreonam-avibactam combination appears to be active against carbapenem-resistant *Stenotrophomonas maltophilia* and *Burkholderia cepacia* complex.^40^

In the study, the combination of CAZ-AVI with aztreonam proved to be effective in treating GNB that co-produces metallo and other class A beta-lactamases. CAZ-AVI combined with aztreonam showed to be synergistic by disc approximation testing and proved to be clinically efficacious in treating a patient with a disease caused by SPM-1 producing *Pseudomonas aeruginosa*. This combination also proved to be a therapeutic option for infection caused by *S. maltophilia*. Falcone et al. demonstrated higher rates of therapeutic success, lower mortality and lower nephrotoxicity in the treatment of BSI in adults caused by metallo-beta lactamase-producing *Enterobacterales* with the combination CAZ-AVI and aztreonam when compared to regimens using other antibiotics showing in vitro activity such as polymyxin B, aminoglycosides, fosfomycin and tigecycline.^16^^,^^41^

The use of CAZ-AVI in the treatment of ventriculitis due to *K. pneumoniae* in a 7-year-old patient showed a good therapeutic response, expanding the possibilities of using the medication. Despite the negative cultures even after prior administration of intravenous and intrathecal polymyxin B, the patient-maintained CSF altered parameters and persistent drowsiness. After switching to CAZ-AVI, there was progressive clinical and laboratory improvement. Case series have demonstrated success in the treatment of CNS infection caused by Enterobacteriaceae and *Pseudomonas aeruginosa* MDR with ceftazidime-avibactam in adults and children. Although robust data are limited, recent studies suggest that there is approximately 38 % penetration of CAZ-AVI in the CNS, levels sufficient for its bactericidal action.^42^, ^43^, ^44^

The patient with endocarditis had a good clinical evolution with the use of two weeks of CAZ-AZI and after his stabilization. CAZ-AVI was maintained in combination with polymyxin B and meropenem for two more weeks resulting in clinical cure. He kept progressively improving ultrasound image and anti-coagulation due to the associated hypothesis of thrombus. Data on its use in the treatment of infective endocarditis is scarce, restricted to case reports in adults.^45^

A systematic review of CAZ-AVI use in severe MDR GNB infections in adults highlights its effectiveness in a landscape of rising resistance and limited therapeutic options. However, more data on its pharmacodynamics are needed to refine its use across different patient profiles and types of infections.^46^

The study revealed two unrelated deaths that were not attributed to the bacterial infection or medication and no other adverse events related to the use of the antimicrobial were seen, such as gastrointestinal complaints, rash and other altered laboratory parameters.

In the case series, it was possible to observe that patients often presented hypoalbuminemia (serum albumin less than 3g/dL) and an increase in the estimated glomerular filtration rate with CrCl above 130 mL/min/1.73m^2^, common findings in oncology and critically ill patients.^47^ Although one study found usual dose might achieved adequate pharmacodynamic target in children older than 3-months, further research is necessary to set up the best antimicrobial combination as well as pK/pD parameters for optimization of antimicrobial regimens in critical ill children.^48^

All patients responded to the CAZ-AVI therapy. Despite being a relatively new antibiotic, resistance to CAZ-AVI has already been described. The most common mechanisms involve the co-existence of carbapenemases such as metallocarbapenemase or other class D oxacillinases such as Oxa-24/40. Other mechanisms may be due to hyperproduction of extended spectrum beta-lactamase such as VEB (Vietnamese Extended-spectrum Beta-lactamase) and carbapenemases, gene mutation Ex: KPC-2 omega loop mutation. In non-fermenting BGN such as *Pseudomonas aeruginosa*, resistance can additionally arise from mutation in genes encoding beta-lactamases such as ampC, increase in the efflux system and entry deficiency due to reduction of porins.^49^

In life-threatening infections due to MDR or XDR GNB, studies suggest a possible benefit of combining CAZ-AVI with other antibiotics. The combination of CAZ-AVI with meropenem, amikacin and aztreonam appears to significantly reduce the MIC for ceftazidime up to fourfold in MDR strains of *Klebsiella pneumoniae* and in the case of *Pseudomonas aeruginosa*, the combination with meropenem or colistin has shown a similar, albeit less pronounced, effect.^50^

This study presents some limitations as a retrospective observational study with few subjects, limited microbiologic investigation and access to CAZ-AVI at the period also led to previous selection of patients with documented GNB infection that can arise risk of bias and caution with interpretation of results. Nonetheless, this study exposes a real-life scenario in developing countries and challenges in treatment of MDR BGN infection in children with cancer. Molecular resistance mechanisms were done only in blood culture, and other limitations of this study were considered

## Conclusion

We reported our experience with 13 courses of CAZ-AVI to treat infection by BGN in oncologic patients. In this cohort (92 %) recovered and achieved microbiological cure. No adverse events were attributed. CAZ-AVI may be a choice for treating BGN infections in the pediatric cancer setting.

## Conflicts of interest

The authors declare no conflicts of interest.
